# Characterization of the Chemopreventive Properties of *Cannabis sativa* L. Inflorescences from Monoecious Cultivars Grown in Central Italy

**DOI:** 10.3390/plants12223814

**Published:** 2023-11-09

**Authors:** Silvia Di Giacomo, Ester Percaccio, Annabella Vitalone, Cinzia Ingallina, Luisa Mannina, Alberto Macone, Antonella Di Sotto

**Affiliations:** 1 Department of Physiology and Pharmacology “V. Erspamer”, Sapienza University of Rome, P.le Aldo Moro 5, 00185 Rome, Italy; ester.percaccio@uniroma1.it (E.P.); annabella.vitalone@uniroma1.it (A.V.); 2 Unit of Human Nutrition and Health, Department of Food Safety, Nutrition and Veterinary Public Health, National Institute of Health, 00161 Rome, Italy; 3 Department of Drug Chemistry and Technologies, Sapienza University of Rome, P.le Aldo Moro 5, 00185 Rome, Italy; cinzia.ingallina@uniroma1.it (C.I.); luisa.mannina@uniroma1.it (L.M.); 4 Department of Biochemical Science “A. Rossi Fanelli”, Sapienza University of Rome, P.le Aldo Moro 5, 00185 Rome, Italy; alberto.macone@uniroma1.it

**Keywords:** hemp by-products, inflorescences, terpenoids, entourage effects, antimutagenic activity, cytotoxicity, glucose-6-phosphate dehydrogenase, lactate dehydrogenase, oxidative stress

## Abstract

Hemp bioproducts hold great promise as valuable materials for nutraceutical and pharmaceutical applications due to their diverse bioactive compounds and potential health benefits. In line with this interest and in an attempt to valorize the Lazio Region crops, this present study investigated chemically characterized hydroalcoholic and organic extracts, obtained from the inflorescences of locally cultivated Felina 32, USO 31, Ferimon and Fedora 17 hemp varieties. In order to highlight the possible chemopreventive power of the tested samples, a bioactivity screening was performed, which included studying the antimutagenic activity, radical scavenging power, cytotoxicity in human hepatoma HepG2 cells, leakage of lactate dehydrogenase (LDH) and modulation of the oxidative stress parameters and glucose-6-phosphate dehydrogenase (G6PDH) involved in the regulation of the cell transformation and cancer proliferation. Tolerability studies in noncancerous H69 cholangiocytes were performed, too. The organic extracts showed moderate to strong antimutagenic activities and a marked cytotoxicity in the HepG2 cells, associated with an increased oxidative stress and LDH release, and to a G6PDH modulation. The hydroalcoholic extracts mainly exhibited radical scavenging properties with weak or null activities in the other assays. The extracts were usually well-tolerated in H69 cells, except for the highest concentrations which impaired cell viability, likely due to an increased oxidative stress. The obtained results suggest a possibility in the inflorescences from the Felina 32, USO 31, Ferimon and Fedora 17 hemp varieties as source of bioactive compounds endowed with genoprotective and chemopreventive properties that could be harnessed as preventive or adjuvant healing strategies.

## 1. Introduction

Industrial hemp, scientifically known as *Cannabis sativa* L., has emerged as a valuable and versatile plant for a wide array of conventional and advanced industrial products [1,2]. It is characterized by a low content of the psychoactive cannabinoid Δ9-tetrahydrocannabinol (THC), which distinguishes it from the drug-type crops which are mainly cultivated for recreational or medicinal use [3]. The cultivation of industrial hemp dates back centuries worldwide, especially in Europe and has become popular owing to the adaptability to various climates and soils; moreover, it has been shown to improve soil health and biodiversity, making it a sustainable strategy for adapting eco-friendly practices [1,4,5].

In the industrial sector, hemp is renowned for its strong and versatile fibers, used in the production of textiles, ropes and construction materials [6]. Additionally, hemp seeds are rich in healthy fats, proteins and essential nutrients, making them a valuable component in the food and dietary supplement industries; hemp-derived biodegradable plastics and biofuels have been developed, too [6]. 

Hemp is recognized to be a rich source of diverse bioactive compounds, thus holding promise for pharmaceutical, nutraceutical and medicinal applications [7]. A great deal of attention has been especially devoted to phytocannabinoids, among which cannabidiol was found to possess multiple bioactivities, such as anti-inflammatory, analgesic, anxiolytic and neuroprotective effects [8]. Moreover, other groups of phytochemicals, such as monoterpenes, sesquiterpenes and flavonoids have been highlighted as possessing interesting bioactivities [7,9,10,11], thus suggesting their contribution to those of the entire hemp phytocomplex. Indeed, emerging evidence suggests the occurrence of entourage effects among the phytochemicals of the hemp phytocomplexes, which can lead to both synergistic and antagonistic interactions, which may enhance or modify the biological effects of the isolated compounds, as well as limiting the toxic effects [12,13,14].

Being a versatile crop, hemp comes in various varieties, each displaying unique agronomic and phytochemicals characteristics which pose challenges to optimizing their use for specific applications in the industrial, medicinal or nutritional fields [15]. For instance, Futura 75 is mainly cultivated to produce seeds, fibers and high yields of essential oil, while Fedora is mainly cultivated to obtain seeds and oil [16]. The seed oil from Ferimon, Fedora and Felina 32 were found to be enriched with omega-6 fatty acids [17]. Furthermore, Carmagnola and USO 31 are sources of strong and durable fibers [18]. Other by-products from these varieties, such as leaves, inflorescences and essential oils, have been also studied in order to valorize the hemp waste materials and to exploit them for industrial and pharmaceutical purposes [13,19,20,21,22].

In line with this evidence and in the attempt to highlight possible healing properties of valuable by-products from hemp varieties, in the present study, we investigated the bioactivity profile of some extracts that come from the inflorescences of Felina 32, USO 31, Ferimon and Fedora 17 hemp varieties, cultivated in the Lazio Region according to the current law [23]. In our previous study, a peculiar phytochemical composition of these varieties, composed of phytocannabinoids, sesquiterpenes, polyphenols and flavonoids, has been characterized over the seasons [23], thus suggesting a possible diverse application in industrial and/or pharmaceutical/nutraceutical fields. 

To this end, in this study we evaluated the antimutagenic activity in the bacterial reverse mutation assay and the antioxidant power of both the organic and hydroalcoholic extracts from the inflorescence of Felina 32, USO 31, Ferimon and Fedora 17 hemp varieties, collected in June and September, according to previous methods [24,25]. Moreover, the cytotoxicity of the tested samples in human hepatoma cell line HepG2; the leakage of the cytoplasmic lactate dehydrogenase (LDH) enzyme [26]; and the modulation of the glucose-6-phosphate dehydrogenase (G6PDH) enzyme, involved in the pentose phosphate pathway (PPP) and in the regulation of the cell transformation and cancer proliferation [27], were investigated. The effects of the treatments on the intracellular oxidative stress parameters, i.e., reactive oxygen species (ROS) and glutathione levels, were determined according to previous studies [28]. Lastly, tolerability studies in human H69 cholangiocytes were also performed.

## 2. Results

### 2.1. Antimutagenic Activity in Bacterial Reverse Mutation Assay

The potential antimutagenicity of the extracts against the oxidative mutagen tBOOH (40 µg/mL) was evaluated at the concentrations of 50, 100 and 250 μg/mL, which were found to be nontoxic and lacking in mutagenic effects in preliminary assays. Also, the antioxidant agent Trolox was included as positive control for the antimutagenic activity. At a concentration of 30 μg/mL, it produced about a 60% inhibition of the tBOOH-mutagenicity: this effect was classified as strong, according to Di Sotto et al. [25]. Under our experimental conditions, the tBOOH significantly increased the WP2uvrAR revertant colonies (about 5–6 fold) with respect to the vehicle control.

**Figure 1:**
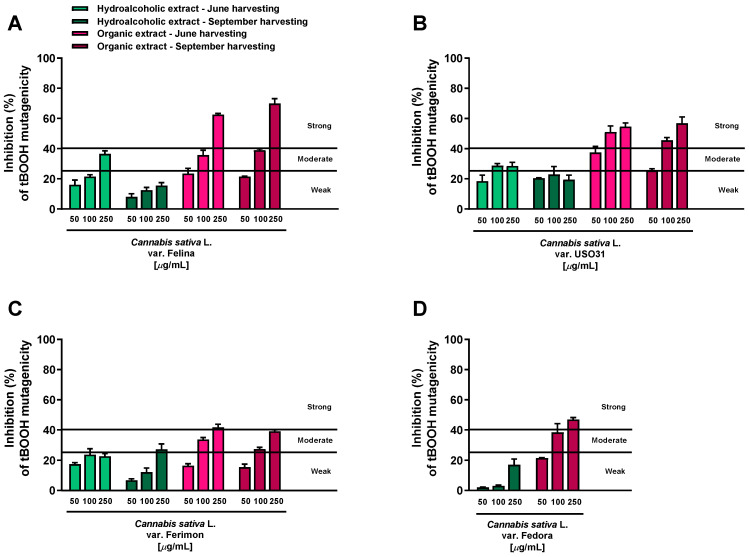
Inhibition of the tert-butyl hydroperoxide (tBOOH) mutagenicity (40 μg/mL) by hemp extracts obtained from the Monoecious Cultivars inflorescences collected in June and September in the WP2uvrAR strain. Values represent the mean ± SEM (*n* = 6). (**A**) *C. sativa* var. Felina 32; (**B**) *C. sativa* var. USO31; (**C**) *C. sativa* var. Ferimon; (**D**) *C. sativa* var. Fedora.

All the extracts were found able to interfere with the tBOOH-induced mutagenicity, although with different potency and effectiveness. According to the classification described by Di Sotto et al. [25], the hydroalcoholic samples obtained from both June and September harvesting produced weak (<25% inhibition) or moderate (inhibition from 25 to 40%) inhibitory effects, while the antimutagenicity of the organic extracts was generally moderate and strong (>40% inhibition) (Figure 1). 

Among the hydroalcoholic samples, the extracts from the June-harvested inflorescences of Felina and USO31 varieties were the most effective. At the highest concentration tested (250 μg/mL), their antimutagenicity was 36.5% and 31.5%, respectively (Figure 1A,B); in all the other cases, a maximum inhibition from 15.6% to 27% was reached (Figure 1A–D). Conversely, strong antimutagenic effects were obtained when the WP2uvrAR strain was treated with the organic extracts of all varieties at both harvesting periods: the maximum inhibitions of about 62.5% and 70% were reached at the highest concentrations of the Felina samples from June and September harvesting, respectively (Figure 1A). Similarly, the extracts from the USO31 inflorescences collected in June and September produced the maximum inhibitions of 55% and 57% (Figure 1B). A lower but always strong antimutagenic effect was obtained using the organic samples of Ferimon (maximum inhibition of about 40%) and Fedora (up to 47% inhibition) (Figure 1C,D).

On the basis of this evidence, the tested extracts displayed antimutagenic properties against the oxidative mutagen tBOOH, although with some different potency and efficacy profiles. 

### 2.2. Antioxidant Activity

**Figure 2:**
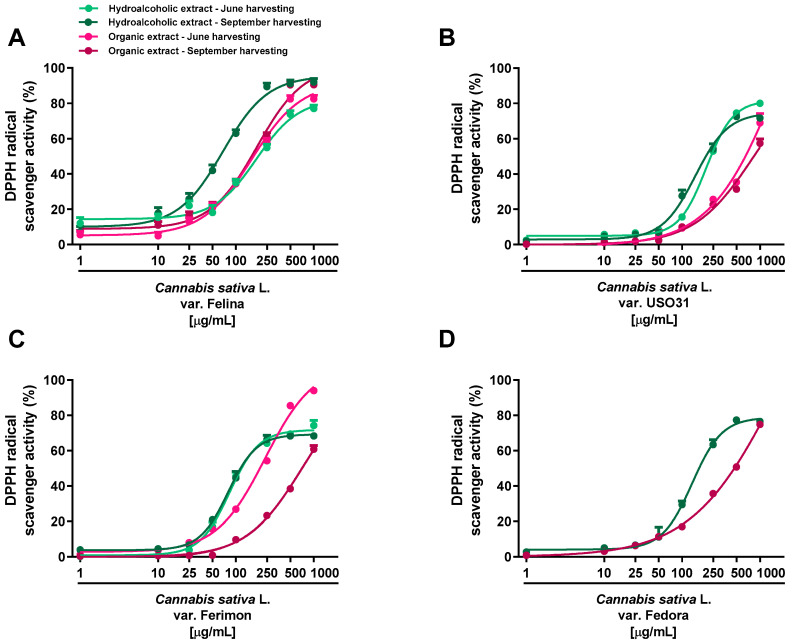
Scavenger activity of the hemp extracts obtained from the Monoecious Cultivars inflorescences collected in June and September against the DPPH radical. (**A**) *C. sativa* var. Felina 32; (**B**) *C. sativa* var. USO31; (**C**) *C. sativa* var. Ferimon; (**D**) *C. sativa* var. Fedora. The values represent the mean ± SEM (*n* = 6).

**Figure 3:**
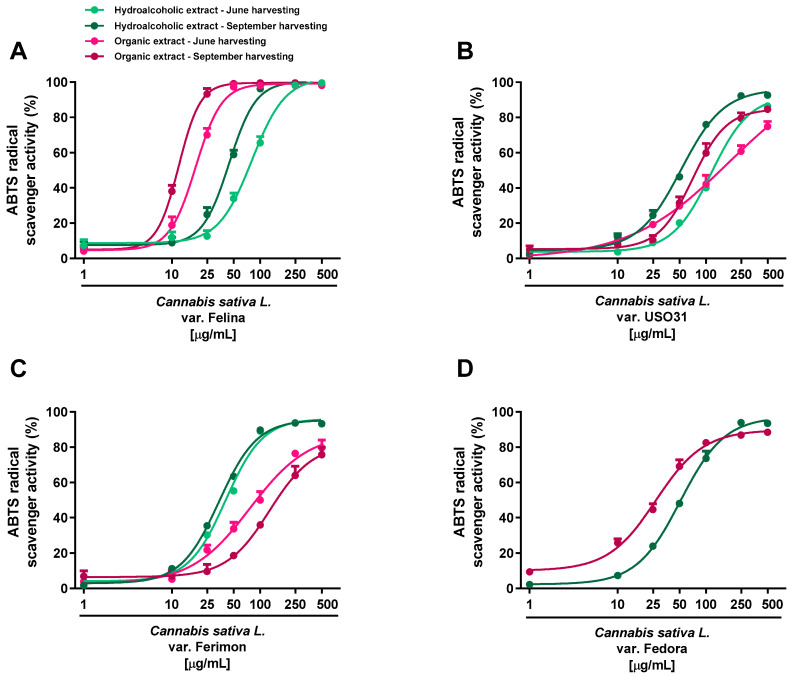
Scavenger activity of hemp extracts obtained from the Monoecious Cultivars inflorescences collected in June and September against the ABTS radical. (**A**) *C. sativa* var. Felina 32; (**B**) *C. sativa* var. USO31; (**C**) *C. sativa* var. Ferimon; (**D**) *C. sativa* var. Fedora. The values represent the mean ± SEM (*n* = 6).

Under our experimental systems, the extracts were found able to interfere, in a significant and concentration-dependent manner, with both DPPH and ABTS radicals, although with a different potency and efficacy (Figure 2 and Figure 3; Table 1 and Table 2). In the DPPH assay, the hydroalcoholic extracts obtained from all the varieties displayed higher potency with respect to the organic ones (Figure 2).

Significant potency differences were also related to the harvesting periods, with the samples from September being the most potent (Table 1 and Table 2). Among the varieties, the hydroalcoholic samples from Ferimon and Felina exhibited the higher radical scavenger potency, because they were able to neutralize DPPH at the lowest concentrations (maximum inhibition at 250 and 500 μg/mL). For the hydroalcoholic samples from June, Ferimon displayed an IC50 value of about 2-fold lower than that of Felina (Table 1). At the September harvesting, these varieties exhibited similar potency; however, a higher potency with respect to the hydroalcoholic extract from June was found for Felina, with lowered IC50 values of about 2.7 (Table 2). Regarding Fedora and USO31 varieties, the DPPH radical scavenging effect of the hydroalcoholic samples occurred at higher concentrations, thus resulting in a 2- and 3-fold lower potency (Table 2). The scavenging power of the organic extracts occurred generally at high concentrations: an IC50 ratio between organic and hydroalcoholic extracts from about 2- to 10-fold was highlighted, thus suggesting the highest potency of the hydroalcoholic samples (Table 1 and Table 2). Felina was the most potent variety as the IC50 was from 3- to 6-fold lower than that of Fedora, Ferimon and USO31.

Regarding the ABTS assay, all the extracts displayed antioxidant effects at low concentrations; the maximum radical scavenging effect (at least 90% inhibition) of both hydroalcoholic and organic samples reached from 50 to 500 μg/mL (Figure 3; Table 1 and Table 2).

Significant potency differences were also related to the harvesting periods, with the sample from September being the most potent (Table 1 and Table 2). According to the statistical analysis, the IC50 values of Felina 32 organic samples from the September harvest were significantly (*p* < 0.01) lower than those of the other varieties, thus suggesting that Felina 32 has the highest potency as a radical scavenger product. Indeed, Fedora, Ferimon and USO31 were found to be of lower potency, as their IC50 values increased from about 2- to 10-fold with respect to Felina’s values. Conversely, no statistical differences were highlighted among the hydroalcoholic extracts of the different varieties. Accordingly, the Felina 32 organic extract from the June-harvested samples was the most potent scavenger, as its IC50 value was 4- and 7-fold lower with respect to the values of Ferimon and USO31, respectively. Among hydroalcoholic extracts, Ferimon stood out as its IC50 values were 2- and 3-fold higher than those of Felina 32 and USO31 2, respectively. The positive control Trolox was more potent than the tested extracts, with the IC50 values recorded as 5.0 (4.4–5.8) and 2.1 (1.6–2.4) μg/mL against the DPPH and ABTS radicals.

### 2.3. Antiproliferative Activity in Human Hepatoma HepG2 Cells

**Figure 4:**
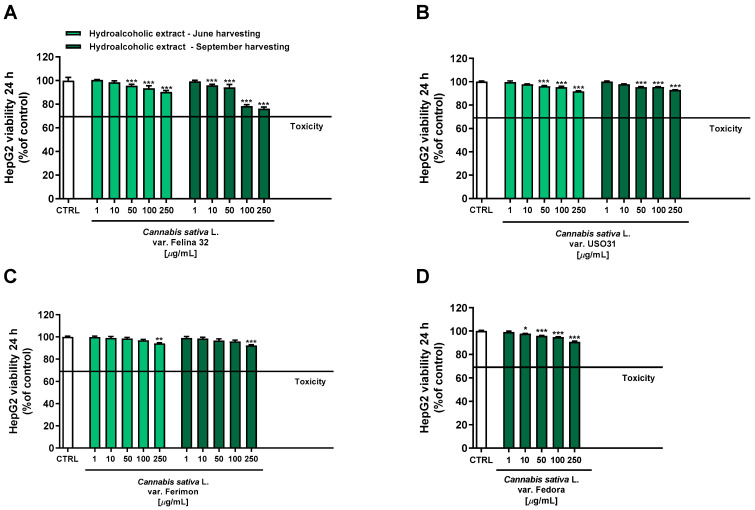
Cytotoxicity of hydroalcoholic extracts obtained from the June- and September-harvested inflorescences of the different varieties of *Cannabis sativa* L in human hepatoma HepG2 cells determined by using the MTT assay after 24 h of exposure. (**A**) *C. sativa* var. Felina 32; (**B**) *C. sativa* var. USO31; (**C**) *C. sativa* var. Ferimon; (**D**) *C. sativa* var. Fedora. The data are displayed as mean ± SE of at least three independent experiments (*n* = 3). * *p* < 0.05, ** *p* < 0.01, and *** *p* < 0.001 vs. control (one-way ANOVA followed by Dunnett’s multiple comparison post-test).

Under our experimental conditions, hydroalcoholic extracts from both June and September harvesting showed a scant antiproliferative activity toward the human hepatoma cells HepG2 after 24 h of treatment. Indeed, all the extracts exhibited a maximum cytotoxicity of 10% at the highest tested concentration of 250 μg/mL, except for the hydroalcoholic extract of Felina 32 from the September harvesting which lowered the HepG2 cell viability to about 24% (Figure 4).

**Figure 5:**
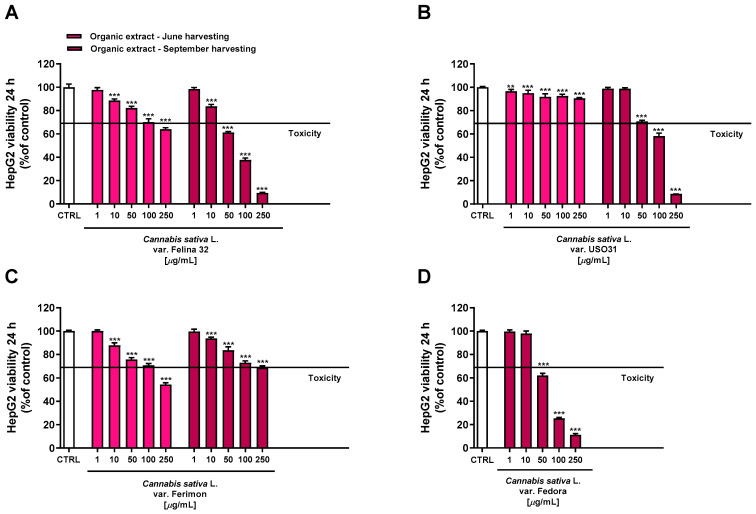
Cytotoxicity of the organic extracts obtained from the June- and September-harvested inflorescences of the different varieties of *Cannabis sativa* L in human hepatoma HepG2 cells determined using MTT assay after 24 h of exposure. (**A**) *C. sativa* var. Felina 32; (**B**) *C. sativa* var. USO31; (**C**) *C. sativa* var. Ferimon; (**D**) *C. sativa* var. Fedora. The data are displayed as mean ± SE of at least three independent experiments (*n* = 3). ** *p* < 0.01 and *** *p* < 0.001 vs. control (one-way ANOVA followed by Dunnett’s multiple comparison post-test).

Conversely, the organic extracts showed a more marked cytotoxicity, with the extracts from September being the most potent (Figure 5). Regarding the June-harvesting samples, Felina 32 and Ferimon were determined to have, at the highest tested concentration, a maximum reduction in cell viability of about 36% and 46%, respectively, while only a 10% cytotoxicity was highlighted for USO31. 

Regarding the September-harvested samples, Felina 32 and Fedora were shown to be the most effective due to a reduction in cell viability of about 62% and 75% at the concentration of 100 μg/mL, while USO31 and Ferimon showed only 42% and 27% of cytotoxicity. The following IC50 values with the relative confidence limits (CL) were calculated: 67.7 (CL, 64.5–71.0) μg/mL for Felina 32, 101.6 (CL 95.6–108.1) μg/mL for USO31 and 61.5 (58.4–64.7) μg/mL for Fedora. Unfortunately, it was not possible to calculate the IC50 value for the Ferimon organic sample, as it achieved an inhibition lower than 80%. Under our experimental conditions, the IC50 value of the positive control doxorubicin was 18.7 (13.8–21.3) μg/mL.

**Figure 6:**
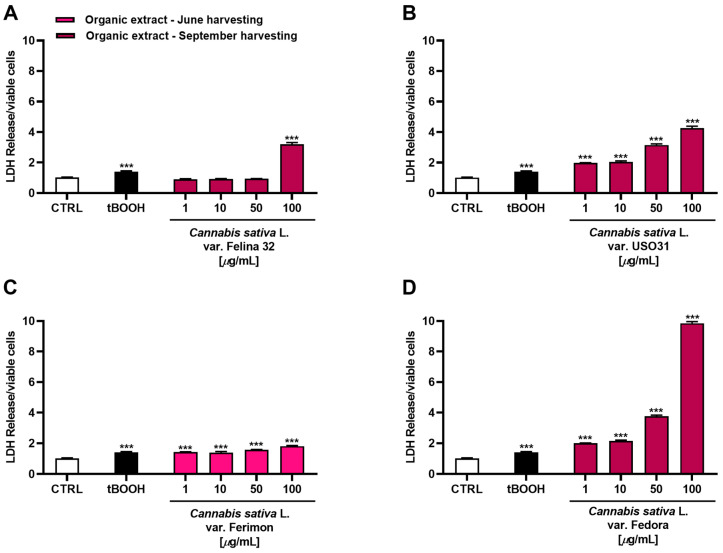
Cytotoxicity of organic extracts obtained from the inflorescences of different varieties of *Cannabis sativa* L in human hepatoma HepG2 cells determined by LDH release assay after 24 h of exposure. (**A**) *C. sativa* var. Felina 32 organic extract from September harvesting; (**B**) *C. sativa* var. USO31 organic extract from September harvesting; (**C**) *C. sativa* var. Ferimon organic extract from June harvesting; (**D**) *C. sativa* var. Fedora organic extract from September harvesting. The data are displayed as mean ± SE of at least three independent experiments (*n* = 3). *** *p* < 0.001 vs. control (one-way ANOVA followed by Dunnett’s multiple comparison post-test).

Considering that the highest cytotoxicity was exhibited by the organic samples of Felina 32, USO31 and Fedora from the September harvest and by that of Ferimon from the June harvest, we carried out the Lactate dehydrogenase (LDH) release assay to evaluate the ability of the treatments to damage cell membrane, which is useful as an indicator of necrosis occurrence. Under our experimental conditions, all the samples were able to induce LDH release although with some differences (Figure 6). Particularly, the Felina 32 organic extract determined a cell membrane damage only at the highest tested concentration of 100 μg/mL. Conversely, the other varieties were effective starting from the lowest concentration, although the maximum effect was always reached at the highest concentrations. The Ferimon organic extract from the June harvesting was the least cytotoxic. By comparison, the Fedora sample was the most toxic, determining an effect about 3-, 2-, and 5-fold higher than Felina 32, USO31, and Ferimon, respectively, at the concentration of 100 μg/mL.

Based on the present results, the concentration range of 1–100 µg/mL was chosen to be tested in the subsequent studies, which were aimed at evaluating the mechanism underlying the observed cytotoxic effect for the organic extracts of Felina 32, USO31 and Fedora from the September harvest and Ferimon from the June harvest.

### 2.4. Inhibition of G6PDH in Human Hepatoma HepG2 Cells

The glucose-6-phosphate dehydrogenase (G6PDH) is a rate-limiting enzyme in the pentose phosphate pathway (PPP), which has been implicated in the regulation of cell transformation, proliferation, apoptosis and angiogenesis in several kinds of tumors, including liver cancer [29]. Therefore, we have evaluated the ability of our samples to inhibit this enzyme as a potential mechanism of their antiproliferative activity. In particular, we tested the concentration of 50 µg/mL, which produced a higher than 10% reduction in cell viability for the organic samples. 

**Figure 7:**
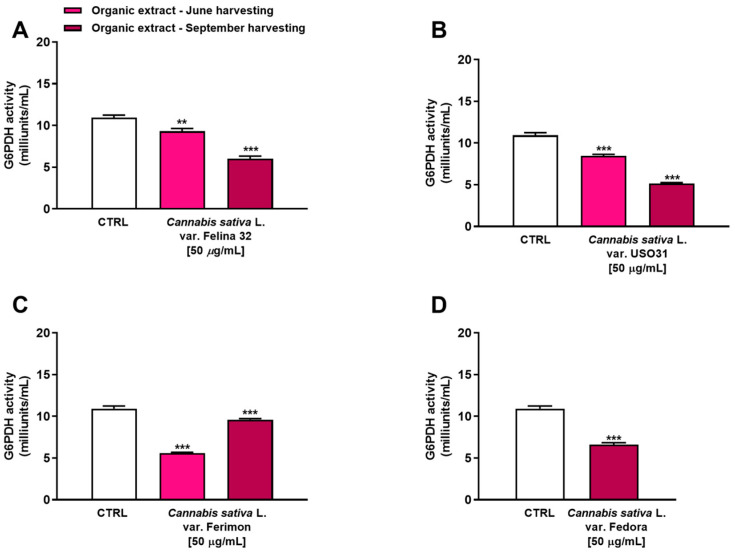
Inhibition of glucose-6-phosphate dehydrogenase (G6PDH) activity by the organic extracts from (**A**) *C. sativa* var. Felina 32, (**B**) *C. sativa* var. USO31, (**C**) *C. sativa* var. Ferimon, and (**D**) *C. sativa* var. Fedora in HepG2 cells. The data are mean ± SE from at least three independent experiments (*n* = 3). ** *p* < 0.01 and *** *p* < 0.001 vs. control (one-way ANOVA followed by Dunnett’s multiple comparison post-test).

Under our experimental conditions, all the samples were able to affect G6PDH activity. In particular, among extracts from the June harvest, the Ferimon cultivar was the most effective as an inhibitor of G6PDH activity by about 2-fold with respect to the control (Figure 7). Conversely, Felina 32 and USO31 slightly affected the enzyme, reducing its activity by about 1.2- and 1.3-fold with respect to the basal level. Interestingly, an inverse trend was highlighted among the samples from the September harvest. Indeed, while Ferimon was the least effective extract as it demonstrated an inhibition of G6PDH activity by only 1.1-fold, Felina 32, USO31 and Fedora showed a higher effect than that of the June harvest. Specifically, they reduced the enzyme activity by about 1.8-, 2.1-, and 1.6-fold, respectively, with respect to the control.

Overall, the present data allowed us to hypothesize that the impairment of G6PDH could be related to the cytotoxicity observed. Thus, in the subsequent experiments, we tried to corroborate our hypothesis by studying the potential increase in oxidative stress and the imbalance of the GSH antioxidant system, which are direct consequences of G6PDH inhibition.

### 2.5. Oxidative Stress Modulation

The levels of ROS were measured by performing the DCFH-DA assay. In particular, a protocol consisting of a 24 h treatment for the samples was applied. Conversely, an exposure schedule of 3 h was used for the positive control tBOOH (500 µM), due to its marked oxidative activity [30].

**Figure 8:**
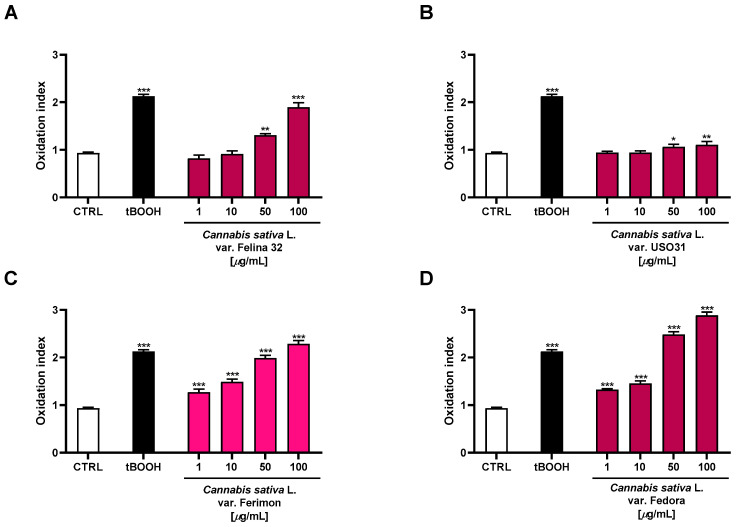
Effects of the organic extracts from the hemp varieties and of the positive control tBOOH (500 μM) on the intracellular ROS levels in HepG2 cells (**A**) *C. sativa* var. Felina 32 organic extract from September harvesting; (**B**) *C. sativa* var. USO31 organic extract from the September harvest; (**C**) *C. sativa* var. Ferimon organic extract from the June harvest; (**D**) *C. sativa* var. Fedora organic extract from September harvesting. ROS levels are expressed as oxidation index with respect to the basal levels. The data are mean ± SE from at least three independent experiments (*n* = 3). * *p* < 0.05, ** *p* < 0.01, and *** *p* < 0.001 vs. control (one-way ANOVA followed by Dunnett’s multiple comparison post-test).

Under our experimental conditions, the oxidative agent tBOOH induced a statistically significant increase in ROS production by about 2-fold with respect to the control. Moreover, the organic extracts from the hemp varieties exhibited significant oxidative properties, with Fedora being the most effective (Figure 8). Indeed, 2.7- and 3-fold increases in ROS production, with respect to the basal, were observed at the highest-tested concentrations, respectively. The Felina 32 and Ferimon samples were also able to induce oxidative stress, reaching values similar to that of the positive control tBOOH at 100 µg/mL (about 2-fold ROS increase). Conversely, the USO31 samples produced only a slight increase in ROS production (about 1.2-fold) at the highest-tested concentrations. 

**Figure 9:**
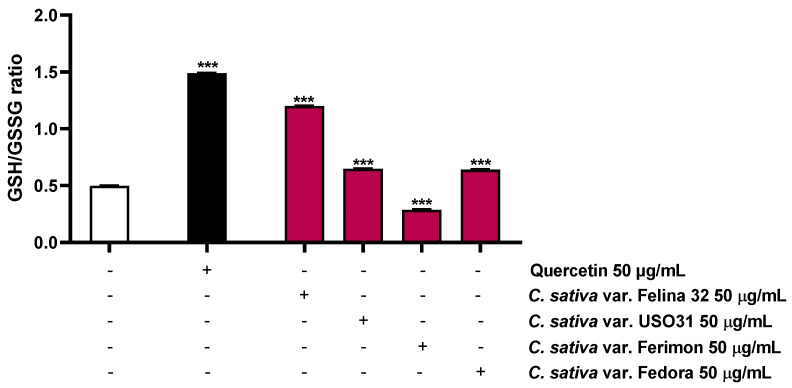
Effects of the positive control quercetin (50 µM) and the organic extracts of Felina 32, USO31 and Fedora from the September harvest and Ferimon from the June harvest (50 µg/mL) on the GSH/GSSG ratio in HepG2 cells. GSH and GSSH were evaluated in cell lysates and calculated with respect to the calibration curves of GSH and GSSG. The data are mean ± SE from at least three independent experiments (*n* = 3). *** *p* < 0.001 vs. tBOOH (one-way ANOVA followed by Dunnett’s multiple comparison post-test).

Considering that the ROS production leads to a depletion in antioxidant reserve, such as reduced glutathione (GSH), the possible modulation of this target by the hemp organic extracts was also investigated. As expected, the positive control quercetin, known for its antioxidant properties [31], highly increased the GSH/GSSG ratio by about 3-fold (Figure 9; Table 3). Conversely, among the hemp samples, only Ferimon was able to reduce the GSH/GSSG ratio by about 1.7-fold. In particular, it induced a slight increase in the GSH levels and almost doubled the GSSG levels with respect to the control (Figure 9; Table 3). Unexpectedly, the other samples raised both the GSH and GSSG concentrations, demonstrating an increase of the GSH/GSSG ratio by about 2.4- and 1.3-fold for Felina 32, USO31 and Fedora, respectively (Figure 9; Table 3).

The present results highlighted the fact that all the samples exerted pro-oxidative effects, although only Ferimon clearly acted by affecting the GSH antioxidant system. Conversely, the other samples seem to stimulate this antioxidant reserve in response to the induced oxidative stress.

### 2.6. Tolerability Studies in Human H69 Noncancerous Cholangiocytes

The extracts were assessed for their tolerability in human intrahepatic noncancerous H69 cholangiocytes, originally derived from a normal liver harvested for transplantation. Bile duct epithelial cells share a common embryological origin with the liver and injuries to these cells may lead to the onset of liver damage. Therefore, they have been chosen to predict the liver tissue’s ability to tolerate the tested hemp extracts. To this end, the cells were exposed to the extracts for 24 h, following the treatment protocols applied to the HepG2 cells. Thereafter, the cell viability and intracellular levels of the reactive oxygen species (ROS) were determined. 

**Figure 10:**
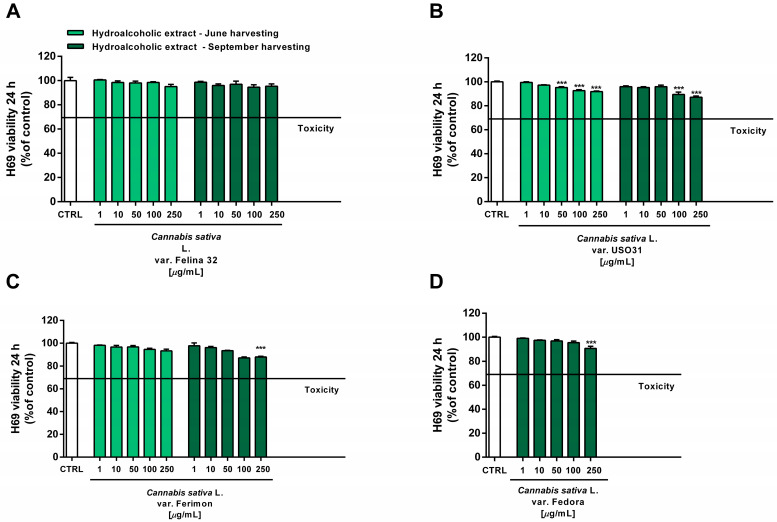
Cytotoxicity of the hydroalcoholic extracts obtained from the June- and September-harvested inflorescences of the different varieties of *Cannabis sativa* L in human H69 noncancerous cholangiocytes determined via MTT assay after 24 h of exposure. (**A**) *C. sativa* var. Felina 32; (**B**) *C. sativa* var. USO31; (**C**) *C. sativa* var. Ferimon; (**D**) *C. sativa* var. Fedora. The data are displayed as mean ± SE of at least three independent experiments (*n* = 3). *** *p* < 0.001 vs. control (one-way ANOVA followed by Dunnett’s multiple comparison post-test).

**Figure 11:**
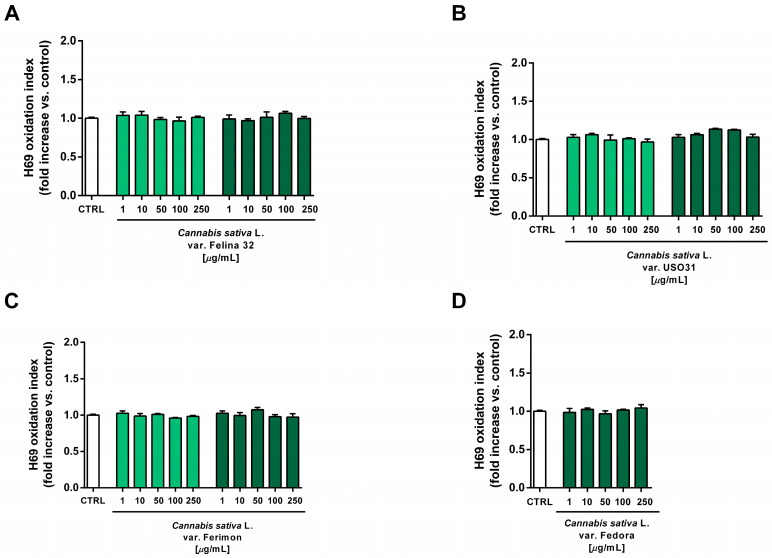
Effects of the hydroalcoholic extracts from the hemp varieties on the intracellular ROS levels in human H69 noncancerous cholangiocytes after 24 h of exposure. (**A**) *C. sativa* var. Felina 32; (**B**) *C. sativa* var. USO31; (**C**) *C. sativa* var. Ferimon; (**D**) *C. sativa* var. Fedora. The data are displayed as mean ± SE of at least three independent experiments (*n* = 3).

**Figure 12:**
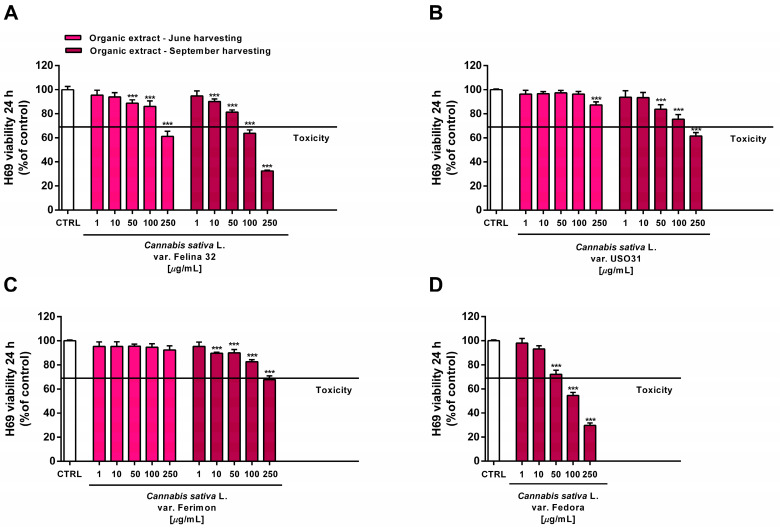
Cytotoxicity of the organic extracts obtained from the June- and September-harvested inflorescences of the different varieties of *Cannabis sativa* L in human H69 noncancerous cholangiocytes determined via MTT assay after 24 h of exposure. (**A**) *C. sativa* var. Felina 32; (**B**) *C. sativa* var. USO31; (**C**) *C. sativa* var. Ferimon; (**D**) *C. sativa* var. Fedora. The data are displayed as mean ± SE of at least three independent experiments (*n* = 3). *** *p* < 0.001 vs. control (one-way ANOVA followed by Dunnett’s multiple comparison post-test).

Under our experimental conditions, the hydroalcoholic extracts from all of the tested crops were found to be devoid of the cytotoxic and pro-oxidant effects contained in H69 noncancerous cells (Figure 10 and Figure 11). Similarly, the organic extract from Felina 32, USO 31 and Ferimon inflorescences harvested in June were nontoxic in the range of the tested concentrations (1–250 µg/mL), except for the highest concentration of the Felina 32 extract, which induced about a 40% decrease in cell viability with respect to control (Figure 12). 

For each crop, the organic extracts from the September harvest were usually more toxic than those harvested in June. Indeed, at the highest concentration of 250 µg/mL they caused a 30% to 60% inhibition in the cell viability: under these conditions, the Felina 32 and Fedora extracts exhibited the highest cytotoxicity, followed by those of USO 31 and Ferimon crops (Figure 12). Slight but significant cytotoxic effects were also observed with 100 µg/mL organic extracts from Felina 32 and Fedora, even though USO 31 and Ferimon exhibited nontoxic effects at the same concentration (Figure 12). However, for all the crops, the IC50 values of both the hydroalcoholic and organic extracts were not evaluable because the observed cytotoxic effect was either null or less than 80% with respect to the control. As expected, the standard anticancer agent doxorubicin exhibited a marked cytotoxic effect in H69 cells with an IC50 value of 15.2 (8.8–28.7) μg/mL.

**Figure 13:**
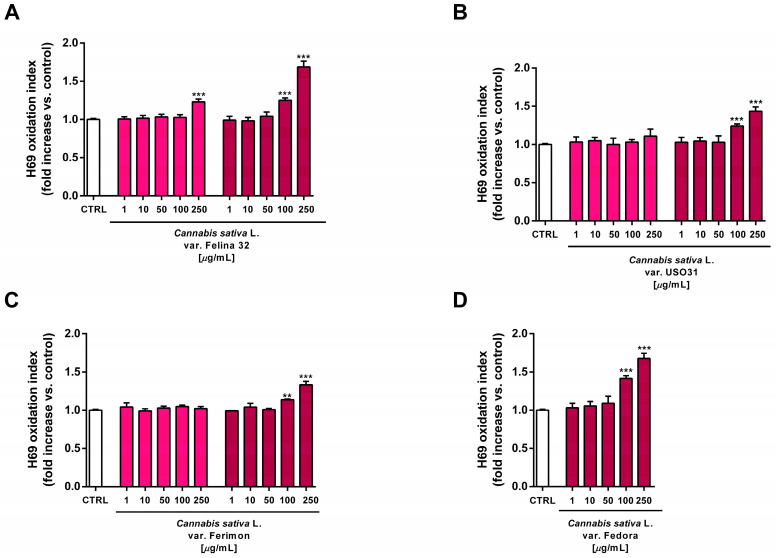
Effects of the organic extracts from hemp varieties on the intracellular ROS levels in human H69 noncancerous cholangiocytes after 24 h of exposure. (**A**) *C. sativa* var. Felina 32; (**B**) *C. sativa* var. USO31; (**C**) *C. sativa* var. Ferimon; (**D**) *C. sativa* var. Fedora. The data are displayed as mean ± SE of at least three independent experiments (*n* = 3). ** *p* < 0.01, and *** *p* < 0.001 vs. control (one-way ANOVA followed by Dunnett’s multiple comparison post-test).

At the highest concentrations of 100 and 250 µg/mL, all the organic extracts from the September harvest also increased the intracellular oxidative stress in H69 cells, suggesting that an oxidative damage can be responsible for the cytotoxic effects of the tested samples (Figure 13). Under the same experimental conditions, the standard prooxidant agent tBOOH (500 μM) induced about a 40% lowering in H69 cell viability, with at least a doubled intracellular level of reactive oxygen species.

## 3. Discussion

In the present study, we investigated the bioactivities profile of the hydroalcoholic and organic extracts obtained through the Bligh-Dyer extractions from the inflorescences of Felina 32, USO 31, Ferimon and Fedora 75 hemp varieties, cultivated in the Lazio Region, in order to highlight their possible healing properties to be further harnessed for pharmaceutical or nutraceutical purposes. 

In our previous study, we particularly demonstrated the phytochemical composition of these cultivars, especially in regard to phytocannabinoids, sesquiterpenes, polyphenols and flavonoids, highlighting the peculiar compositions which could be reflected in different industrial and pharmaceutical applications [23]. In particular, Felina 32 and Fedora 17, especially those inflorescences harvested in September, contained the highest amounts of cannabidiol and cannabidivarin, while cannabinol and cannabichromene were detected in Ferimon, USO 31 and Felina 32. Moreover, USO 31, Felina 32, and Fedora 17 achieved the highest levels of THC. 

Caryophyllane sesquiterpenes were found in all the varieties and increased throughout the season (from July to September); β-caryophyllene achieved the highest levels in Ferimon, Felina 32 and Fedora 17, while β-caryophyllene oxide achieved the highest levels in USO 31, Ferimon and Fedora 17, and humulene in Felina 32, followed by Ferimon, Fedora 17 and Felina 32. Felina 32 and Fedora 17 also contained the sesquiterpenes α- and β-selinene [23]. Regarding phenolic compounds, the highest levels of total polyphenols, tannins and flavonoids were highlighted in the inflorescences of the Felina 32 crop, especially in those harvested in September, followed by USO 31, Fedora 17 and Ferimon [23]. Among the identified phenolics, Felina 32 showed marked levels of catechin and rutin, with lower amounts, albeit higher than those of the other cultivars, of carvacrol, quercetin, coumaric, syringic, chlorogenic and ferulic acids. The other cultivars also contained carvacrol, catechin, rutin and quercetin, albeit at a lower extent than the Felina 32 cultivar. Trans-Cinnamic acid was detected only the USO 31 cultivar, while naringenin and naringin were detected in the Fedora 17 and Ferimon crops, respectively [23].

The rich phytocomplexes of these hemp varieties in regard to phytocannabinoids, terpenes, polyphenols and flavonoids supported our interest in studying their possible chemopreventive properties in terms of antimutagenic activity toward the oxidative damage of tert-butyl hydroperoxide (tBOOH), antioxidant power and cytotoxicity in cancer cells. To further demonstrate the possible mechanisms underlying the cytotoxicity of the extracts, we also evaluated their ability to modulate the intracellular redox state as well as the release of lactate dehydrogenase (LDH) and their expression of the glucose-6-phosphate dehydrogenase (G6PDH). 

The concept of chemoprevention refers to the use of both synthetic and natural compounds to interfere with multiple pathways and processes at different stages of carcinogenesis (initiation, promotion and progression), thus acting as blocking and suppressing agents [32,33]. Blocking agents may directly neutralize carcinogens and reactive species, thus preventing their uptake or their ability to introduce damage into cells, or contribute to their ability to activate cellular defenses, thus repairing possible damages induced by a genotoxic, oxidative and/or inflammatory injury and restoring the cell’s normal functioning [33]. Other chemopreventive agents can impede the development and progression of cancer through several mechanisms, including modulating tumor-promoting and suppressive signalings, promoting cell senescence and blocking the cell cycle or activating the apoptotic cell death, thus acting as suppressing agents [33]. 

Under our experimental conditions, the tested extracts displayed antimutagenic properties against the oxidative DNA damage induced by the mutagen tBOOH, although with different potency and efficacy profiles. The genotoxicity of the tBOOH arises as a result of transition-metal-driven reactions (i.e., iron or copper), which leads to the generation of DNA-reactive free radicals, mainly alkoxyl and peroxyl radicals and leading to lipid, protein and DNA damages [34]. These radicals are soluble into biomembrane bilayers and can start the lipoperoxidation process, whose by-products of highly reactive carbon-based radicals and aldehydes in turn attack cellular structures and DNA and cause widespread cell injury [34,35]. Base substitution mutations via oxidative or alkylative DNA modification and DNA breaks have been found to be associated with tBOOH genetic damage in different in vitro experimental models [36,37,38,39,40].

The interference of the tested samples with the mutagenicity of tBOOH suggests the presence of antioxidant agents in the extract, which are responsible for the neutralization of the ROS species and the prevention of DNA damage. These mechanisms of antimutagenicity are typical of desmutagenic substances, acting as free radical scavengers and/or enzyme inactivators [36], thus either interfering with the formation of active molecules or inducing the inactivation and excretion of toxic species. Our hypothesis is also consistent with the cotreatment protocol here applied, which allows for the suitable identification of desmutagenic agents which can prevent the oxidative damage produced by the reactive species released after the mutagen uptake into the cells, with direct antioxidant or genoprotective effects. 

Based on the literature, a possible antioxidant and antimutagenic role of polyphenols, which represent widely occurring phytoconstituents of *C. sativa* inflorescences, can be hypothesized. In particular, flavonoids such as rutin have been found able to counteract the tBOOH- induced DNA damage [41,42]; moreover, catechin exhibited interesting genoprotective properties [43,44]. Rutin and catechin were mainly concentrated in the hydroalcoholic extracts from the tested hemp crops, achieving the highest levels in the Felina 32 sample from September [23]. Moreover, phenolic acids, such as o-coumaric acid, p-coumaric acid, chlorogenic acid and ferulic acids, were reported to possess antimutagenic properties, likely due to their antioxidant powers and to other desmutagenic mechanisms, such as chemical interactions with the mutagenic species [45,46,47]. Different phenolic acids were identified in the hydroalcoholic extracts of the tested hemp crops, although their levels and identified molecules exhibited significant variation both between the crops and with respect to the harvesting period [23]. For instance, chlorogenic acid was mainly concentrated in the Felina 32 hydroalcoholic extract from the June harvest, while o-coumaric acid was mainly concentrated in the USO 31 and Felina 32 hydroalcoholic extracts from the June and September harvests, respectively [23]. Based on this evidence, the highlighted antimutagenic properties of these extracts seem likely to be due to the subtle interactions occurring within the hemp phytocomplexes among both diverse classes of compounds and diverse compounds in the same groups: these interactions can be considered as a kind of inter- and intra-entourage effect [48]. Furthermore, the possible contributions of other unidentified compounds cannot be excluded.

Regarding the organic extracts, it is likely, based on the composition of our samples [23], that sesquiterpenes and other terpenes may play a crucial role in the antimutagenic properties. Indeed, these extracts were mainly characterized by the presence of caryophyllene sesquiterpenes β-caryophyllene, α-humulene, and their metabolites β-caryophyllene oxide and humulene oxide; among them, β-caryophyllene and β-caryophyllene oxide have been reported to induce antimutagenic and genoprotective effects in different experimental models [49,50,51,52,53]. Among the other identified terpenes [23], the monoterpene carvacrol, detected in a higher amount in the organic extracts of Felina 32 and USO 31, has been highlighted to possess antimutagenic properties, likely ascribable to its ability of modulating membrane lipids and the permeability of its ion channels, or to its antioxidant nature [54]. However, other compounds, such as phytocannabinoids, could contribute to the antimutagenic activity of the tested extracts. Indeed, cannabidiol was reported as being able to protect Caco-2 cells from the DNA damage induced by hydrogen peroxide [55]. Despite this evidence, the differences in the antimutagenicity of the tested organic extracts in relation to their specific composition still require clarification. A more plausible hypothesis is that their effects arise from the contribution of the whole phytocomplex. 

Taking into account the antimutagenicity of the hemp extracts against the oxidative damage induced by tBOOH, we further assessed their radical scavenging abilities, specifically DPPH and ABTS, as a potential direct protective mechanism. Both the hydroalcoholic and organic extracts were endowed with radical scavenging power, albeit usually with a higher potency than that of the first ones, except for the organic extracts of Felina 32 and Fedora 17 hemp varieties against ABTS. The differences in the antioxidant properties highlighted for the tested samples suggest the possible involvement of peculiar bioactive phytoconstituents that are able to differently interfere with DPPH or ABTS species. DPPH is a pre-existing radical with an absorbance which disappears when an electron or hydrogen radical is released from an antioxidant compound, thus forming a stable diamagnetic molecule. Conversely, the ABTS cation, generated just before the experiments through different activators, requires an electron-transfer process to be scavenged and is more reactive than DPPH. Due to their chemical structures, the affinities and kinetics of the antioxidants which react with these radicals can strongly vary, thus resulting in significantly different scavenging potencies [56]. Under our experimental conditions, the IC50 values of the organic extracts against the ABTS radical were from 6- to 16- fold lower than that against DPPH, likely suggesting a major involvement of the electronic transfer in the scavenger activity of these samples. Conversely, the hydroalcoholic samples exhibited a similar power against both radicals, IC50 values from 2- to 4-fold lower against ABTS than DPPH: this evidence suggests the involvement of both electron and hydrogen radical transfer mechanisms. Furthermore, considering that DPPH and ABTS require different alcoholic and aqueous buffers, a contribution of specific bioactive compounds which are soluble in the respective media can be expected. 

Overall, the higher antioxidant activity of the hydroalcoholic extracts with respect to the organic ones is likely ascribable to the presence of the polyphenols and flavonoids such as rutin, quercetin, and catechin, whose radical scavenging power has been well-documented [57,58,59]. Similarly, radical scavenging and antioxidant properties have been reported for carvacrol and β-caryophyllene [60,61,62], suggesting their possible contribution to the activity of the extracts. Moreover, other compounds such as cannabidiol may contribute to the antioxidant activity of the organic extracts. In particular, cannabidiol has been shown to act as an antioxidant, either through interaction with its receptors or in a receptor-independent way. For instance, it can directly scavenge ROS by donating electrons, thereby transforming them into more inert molecules. In addition, it can chelate the transition metals necessary for Fenton reactions, which are behind the non-enzymatic production of ROS [63]. Considering the high amounts of this compound in the inflorescences of Felina 32 and Fedora 17 with respect to those of the other crops, it could contribute to the marked radical scavenging activity of their organic extracts, although the contribution of other compounds in the hemp phytocomplexes cannot be excluded. 

This study also focused on the potential suppressive properties of the hemp extracts in cancer cells. To this end, we used the human hepatoma HepG2 cell line as a model of the gastro-intestinal tract, which can mimic the effect of the samples after oral supplementation and are also endowed with liver metabolic abilities which may activate some bioactive compounds in the hemp phytocomplex. Moreover, highlighting the suppressive effects of the tested samples in this cell model of liver cancer may suggest the need to further explore novel chemopreventive or adjuvant strategies to manage this aggressive and devastating disease. Indeed, until now, most of the studies investigating the antiproliferative activity of hemp extracts have been focused on breast cancer models [13,64,65,66]. Some experiments have been also carried out in liver cancer cells [67]; however, the phytochemical characterization of the samples was mainly focused on cannabinoid content, leaving out other important minor constituents which can greatly contribute to these biological activities. Indeed, it is now recognized that synergistic interactions among cannabis phytochemicals, or the so-called “entourage effect,” are responsible for the wide range of biological activities ascribed to it. Therefore, it is important to perform further studies in order to highlight the possible phytocomplex with a peculiar activity in this highly resistant type of cancer. 

Our findings showed that the organic extracts of all the tested varieties, including the higher potencies of the Felina 32, Fedora and USO 31 varieties from the September harvest, were able to inhibit the cell viability of HepG2 cells, despite lacking effects of the hydroalcoholic extracts; therefore, we selected the active samples for the further mechanistic studies. 

These results agree with our previous study in which the antiproliferative properties of the organic extracts from Felina 32 variety were assessed in human triple negative MDA-MB-468 breast cancer, human epithelial colorectal Caco-2 adenocarcinoma and human bronchoalveolar H358 carcinoma cells, the results of which were especially marked in the first ones [13]. In MDA-MB-468 cells, we also denoted the contribution of caryophyllane sesquiterpenes to the cytotoxicity of Felina 32 phytocomplexes; in particular, these compounds were found to be able to potentiate the effects of cannabidiol (CBD), although their amount was about 100-fold lower than that of CBD. According to this evidence, in the present study, the organic extract of Felina 32 from the September harvest, along with that of Fedora, were the most effective in suppressing the HepG2 cell viability and were also enriched in CBD and caryophyllane sesquiterpenes. However, more complex interactions within the diverse compounds in the same groups (namely intra-entourage effect), and among various groups of phytochemicals (namely inter-entourage effect), such as meroterpenoids, sesquiterpenes and flavonoids, seem to be likely.

Under our experimental conditions, the extracts also induced the leakage of LDH, a cytoplasmic enzyme involved in the glycolytic metabolism of cells; in fact, it is responsible for the conversion of glucose to lactate. Its activity has been found to be especially increased in cancer cells, where it triggers the Warburg effect; it is also released from cells after a membrane damage and consequently increases permeability [68]. The glycolytic metabolism of cancer cells is also controlled by glucose-6-phosphate dehydrogenase, the initial and rate-limiting enzyme in the oxidative phase of the pentose phosphate pathway. It has been traditionally regarded as an antioxidant enzyme, owing to its involvement in the regeneration of NADPH and GSH, which are essential for ROS detoxifying [27]. Moreover, it has been found to be involved in cancer development and in chemoresistance; indeed, an aberrant G6PDH activation has been associated with uncontrolled cell growth in many types of cancers [69].

Our results showed that the organic hemp extracts, especially those from Felina 32, USO 31 and Fedora 17 at the September harvest, were able not only to inhibit the HepG2 cell viability but also to induce the LDH leakage, intracellular ROS production, GSH depletion and inhibition of the G6PDH expression, although at different extents. In fact, the sample from Felina 32 only slightly lowered the GSH levels, while USO 31 produced a weak oxidative stress. Surprisingly, a great oxidative stress and a marked lowering in the GSH were induced by the Ferimon extracts, although with a slight increase in LDH release and low cytotoxicity; these effects were also associated with a weak inhibition of the G6PDH enzyme by the extract from the September harvest. The extract of Fedora 17 induced cytotoxic effects in HepG2 cells, which are associated with a marked ROS increase and LDH leakage. 

These findings suggest that differences in the phytochemistry of each hemp variety may lead to a diverse profile of suppressive abilities in cancer cells. Particularly, the increased ROS levels induced by almost all the organic hemp extracts of September could be ascribed to the presence of β-caryophyllene, which has been reported to induce cytotoxicity in cancer cells via enhancing the oxidative stress [70,71,72]. Similarly, β-caryophyllene oxide and humulene have been found to promote ROS production and lipid peroxidation [73,74,75]. β-Caryophyllene has also been reported to markedly induce the GSH expression in cholangiocarcinoma cells [76], suggesting that it could be involved in the low GSH depletion induced by the Felina 32 extract [76]. According to our hypothesis, the USO 31 crop, which induced low oxidative stress in HepG2 cells, contained lower amounts of β-caryophyllene, β-caryophyllene oxide and humulene [23]. Among the other identified compounds, an increased oxidative stress has also been implicated in the cytotoxicity of cannabidiol [77], suggesting that other compounds in the phytocomplex may contribute to or modulate the effect of caryophyllane sesquiterpenes, leading to the unique activity profile of the tested hemp extracts. 

It must be understood that our study demonstrates the fact that the organic hemp extracts are endowed with antioxidant properties toward radical species and also show oxidative effects in cancer cells. This apparently opposite effect can be explained due to the complex compositions of the extracts, which allow the coexistence of diverse compounds with possible antagonistic effects. Moreover, the same compound could exhibit different properties on the basis of the experimental conditions. In this respect, β-caryophyllene has been reported to be a dual-acting agent, inducing pro-oxidant effects in cancer cells and antioxidant ones in noncancerous cells, thus protecting the cells from the damage of drugs and other toxicants [33,76,78,79].

Under our experimental conditions, we observed that all the tested extracts were generally well-tolerated by H69 cholangiocytes except for the highest concentrations of the organic extracts from the September harvest, particularly those from the Felina 32 and Fedora crops, which induced cytotoxic effects, albeit lower than those in HepG2 liver cancer cells. Accordingly, the extracts were devoid of oxidative effects, except for the highest concentrations of the organic ones (mainly from the September harvest), which induced a moderate increase in the intracellular ROS levels. This may be correlated to the cytotoxicity of the highest concentrations of these extracts. Notably, the inflorescence of Felina 32 and Fedora crops collected in September contained higher levels of cannabidiol than the corresponding samples collected in June as well as the samples collected from other crops. Cannabidiol has been reported to induce cytotoxicity in other noncancerous cell models and to act in a nonspecific manner upon cancer cells [80,81,82], although opposite evidence was reported, too [83,84]. Our samples were less toxic in H69 cells than in HepG2 ones, suggesting that the potential cytotoxicity of cannabidiol may be modulated by other compounds in the hemp phytocomplex, such as β-caryophyllene, leading to an increased tolerability in noncancerous cells. 

Taken together, the obtained results highlight a potential use of the phytocomplexes of the hemp inflorescences for chemopreventive purposes; however, more-in-depth studies are needed to better understand the role of the hemp phytochemical in the observed bioactivities, the mechanisms involved and the efficacy in vivo.

## 4. Materials and Methods

### 4.1. Chemicals

All the substances, including 1,1-diphenyl-2-picrylhydrazyl radical (DPPH), 2,2′-azino-bis (3-thylbenzothiazoline-6-sulfonic acid) diammonium salt (ABTS), 2,2′-azobis (2-methylpropionamidine) dihydrochloride (AAPH), Trolox, tert-butyl hydroperoxide solution (tBOOH; 900 mg/mL), 3-[4,5-dimethylthiazol-2-yl]-2,5-diphenyl tetrazolium bromide (MTT, ≥97.5% purity), 2,7-dichlorofluorescein diacetate and the media for the Ames test, were purchased from Sigma-Aldrich (Milan, Italy). Dulbecco’s Modified Eagle’s Medium (DMEM) was provided by Aurogene (Rome, Italy). Methanol (HPLC-grade), chloroform (HPLC-grade) and ethanol (analytical-grade) were obtained from Carlo Erba Reagenti (Milan, Italy). Double-distilled water was obtained using a Millipore Milli-Q Plus water treatment system (Millipore Bedford Corp., Bedford, MA, USA).

To perform the experiments, all the solutions were prepared in the better solvent, after which they were sterilized and stored at the recommended temperature for a just conservation time. EtOH and DMSO were used at a maximum concentration of 1% *v*/*v* in the cell medium to avoid any cytotoxicity.

### 4.2. Hemp Inflorescences and Extract Preparation

The inflorescences of Felina 32, Ferimon, USO 31 and Fedora 17 hemps, monoecious cultivars of *Cannabis sativa* L. belonging to a cannabidiol (CBD)-rich chemotype, were supplied by the “Canapa Live” cultural association as previously reported [13,23]. The plants were cultivated in experimental fields located in the North Lazio area (Rome, Italy) and harvested at both the early and late flowering stages, namely June and September, except for Fedora 17 which was collected only in September. The sampling was performed in the central part of the cultivation area by picking 30 plants, which were merged to obtain a unique representative sample of each harvesting time. 

The samples were stored at −80 °C before having the Bligh-Dyer extraction applied in order to obtain a hydroalcoholic and an organic extract for each cultivar [13,23]. The flowering aerial parts of each crop, powdered under liquid nitrogen, were briefly mixed with a methanol/chloroform mixture (2:1 *v*/*v*), chloroform, and 1 distilled water to obtain an emulsion, which was stored at 4 °C for 40 min. Subsequently, the sample was centrifuged at 4200× *g* for 15 min at 4 °C, leading to the separation of the hydroalcoholic and organic phases. The pellets were subjected to a second extraction as described above; the obtained extracts were dried under a N_2_ flow at room temperature and stored at −20 °C until analysis. The values of drug extract ratio (DER) are reported by Ingallina et al. [23].

The phytochemical analysis of the extracts highlighted the presence of different polyphenols and terpenes [23]. In regard to the September harvest, Felina 32 was the cultivar with the highest level of total phenolics and tannins, while Fedora 17 had the highest level of total flavonoids. Conversely, in the June samples, the highest content of total phenolics, tannins and flavonoids was found in the hydroalcoholic extracts from the USO 31 crop and in the organic extracts from Felina 32. Catechin, rutin, and quercetin were present in almost all of the hydroalcoholic samples, while carvacrol was ubiquitous. Among the terpenoids, cannabidiol and the caryophyllane sesquiterpenes α-humulene, β-caryophyllene and β-caryophyllene oxide were the most abundant compounds; conversely, the THC level was always under the limit required by the Italian law (max 0.2% *w*/*w*) for industrial hemp [85]. 

### 4.3. DPPH- and ABTS-Free Radical Scavenging Activity Test

The DPPH- and ABTS-radical scavenging activities were determined according to Di Sotto et al. [25] with minor changes. All tests were performed in 96-multiwell microplates away from direct light, and the extracts were tested in the range of 1–1000 µg/mL (dilution factor of about 1:2). The experiments were repeated at least twice, and in each experiment each concentration was tested in triplicate. The data obtained from at least two experiments were pooled for the statistical analysis. In each test, negative or positive controls (Trolox used as the standard antioxidant agent) were included, too. For each test, some wells containing only the test samples were also included, to determine its possible absorbance.

Briefly, a DPPH solution (40 µL; 0.1 mM) was added to the test samples (160 µL), and then the plates were incubated for 30 min in the dark at room temperature. The absorbance of the DPPH radical was measured at 517 nm using a microplate reader (Epoch Microplate Spectrophotometer, BioTeK, Winooski, VT, USA). Regarding the ABTS radical, equal volumes of ABTS (5 mM) and AAPH (2 mM) were mixed and incubated for 45 min at 68 °C to obtain the ABTS radical cation. This solution was diluted in PBS (0.1 M; pH = 7.0) up to an absorbance of about 1.0 at 734 nm. The samples (20 µL) were added to the ABTS radical (180 µL), then the plates were incubated for 10 min at 37 °C in the dark. The absorbance of the ABTS radical was measured at 734 nm by a microplate reader (Epoch Microplate Spectrophotometer, BioTeK). For both assays, the percentage of scavenger activity was calculated as follows: 100 × (A_control_ − A_sample_)/A_control_, where A_control_ is the absorbance of the radical alone, while A_sample_ is that of the radical with the sample.

### 4.4. Antimutagenic Activity

The antimutagenicity of the samples was evaluated using the Ames test (cotreatment protocol) on the *Escherichia coli* WP2*uvr*AR (trpE65Δ*uvr*A pKM101) bacterial strain, kindly supplied by the Research Toxicological Centre (Pomezia, Rome, Italy). The pKM101 plasmid makes the strain error-prone, thereby increasing its susceptibility to mutations induced by cross-linking and pro-oxidant mutagens [86]. The study was carried out according to the previous published method, and preliminary tests were performed in order to find the nontoxic concentrations of the samples and to exclude their possible mutagenic effects [86]. 

In order to evaluate the ability of the extracts to interfere with the oxidative-mediated DNA damage, the pro-oxidant mutagen tert-butyl hydroperoxide (tBOOH; 40 µg/mL) was tested at a concentration producing a submaximal mutagenic effect (about 70% increase in the number of revertant colonies) [25]. Plates containing the vehicle alone or the mutagen represented the lack of mutagenic activity and the maximum mutagenicity, respectively. The possible cytotoxicity of the test samples was evaluated in additional control plates.

The experiments were repeated at least twice, and in each experiment, each concentration was tested in triplicate. The antimutagenicity was considered strong for an inhibition value higher than 40%, and was considered moderate when the inhibition was in the range of 25–40% [25]. When an inhibition lower than 25% occurred, the antimutagenicity was weak or null.

### 4.5. Cell Culture

The human hepatoma cell line HepG2 was obtained from the American Type Culture Collection (ATCC). The SV40-transformed nonmalignant human intrahepatic H69 cholangiocytes were kindly gifted by Romina Mancinelli (Department of Anatomical, Histological, Forensic and Orthopedic Sciences, Sapienza University of Rome, Italy). The HepG2 cells were grown under standard conditions (37 °C and 5% CO_2_), in DMEM medium containing L-glutamine (1% *v*/*v*) and HEPES (15 mM), and were supplemented with 10% heat-inactivated fetal bovine serum (FBS), 100 U/mL penicillin and 100 μg/mL streptomycin. H69 cholangiocytes were cultivated using the typical medium, as previously described [76]. Cells were subcultured every 4 days, renewing the growth medium twice a week, as recommended by the supplier.

### 4.6. Cytotoxicity Assays

The cells were seeded into 96-well microplates (2 × 10^4^ cells/well) and allowed to grow for 24 h. Then, they were treated with progressive concentrations (1, 10, 50, 100, and 250 μg/mL) of the hemp extracts for 24 h. Finally, the cells viability was measured using the MTT assay [87]. The cytotoxicity induced by the treatment was evaluated by comparing the number of viable cells of the vehicle control and that of the treatment. A treatment was considered cytotoxic when the cell viability was less than 70% with respect to the control [87]. Moreover, the most cytotoxic samples were also tested by the Lactate dehydrogenase (LDH) release assay, according to Di Sotto et al. [26], in order to highlight any possible damage at the cell membrane level. The LDH levels were normalized per the number of viable cells. To obtain reproducible data, the assays were carried out at least three times and, in each experiment, each concentration was tested in triplicate, also including a vehicle control. 

### 4.7. Glucose-6-Phosphate Dehydrogenase (G6PD) Activity Assay

The potential inhibition of glucose-6-phosphate dehydrogenase (G6PD) activity was assayed using the Glucose-6-Phosphate Dehydrogenase Kit (Sigma Aldrich, St. Louis, MO, USA, MAK015) according to manufacturer’s instructions. The HepG2 cells were treated with a nontoxic concentration of hemp extracts, namely 50 μg/mL, for 24 h. Then, the cells (1 × 10^6^) were homogenized in equivalent volumes of ice-cold PBS (pH 6.5–8) and centrifuged at 15,000× *g* for 10 min to remove insoluble materials. The supernatants of each sample (50 μL) were added to the master reaction mix (50 μL); afterward, the plate was incubated for 3 min at 37 °C in the dark. The enzyme activity was detected according to the amount of NADH produced through the oxidation of glucose-6-phosphate by measuring the absorbance at λ = 450 nm using a microplate reader (Epoch Microplate Spectrophotometer, BioTeK Instruments Inc., Winooski, VT, USA) at 37 °C. The absorbances were recorded every 5 min for 1 h. The G6PDH activity was calculated by measuring the amount of NADH produced in a 30-min period between 10 min and 40 min after the reaction was begun. To obtain reproducible data, the assay was carried out at least two times and, in each experiment, each concentration was tested in triplicate, also including a vehicle control. Results were expressed as milliunits per milliliter (milliunits/mL), where one unit is the amount of the enzyme that catalyzes the conversion of 1.0 mmole of glucose-6-phosphate to 6-phosphoglucono-d-lactone and generates 1.0 mmole of NADH per minute at 37 °C.

### 4.8. Intracellular Reactive Oxygen Species (ROS) Determination

The 2,7-dichlorofluorescein diacetate assay (DCFH-DA) was performed according to a previous method in order to measure the ROS levels after the treatment with hemp samples [26]. The DCF fluorescence intensity was measured at excitation and emission wavelengths of 485 nm and 535 nm, using a Cytation 1 Cell Imaging Multimode Reader (BioTek, AHSI, Milan, Italy). The results were expressed as a percentage of the negative control after being normalized to viable cells [26].

### 4.9. Determination of Intracellular Glutathione Levels

Intracellular levels of reduced (GSH) and oxidized (GSSG) glutathione were measured using a standardized HPLC-UV method, as previously described [30]. In particular, after the treatment of the HepG2 cells with a subtoxic concentration of hemp extracts (50 μg/mL) for 24 h, the GSH and GSSG levels were evaluated in cell lysates, calculated with respect to the corresponding calibration curves and normalized regarding cell viability. The ratio between the GSH and GSSG was compared among the treatments.

### 4.10. Statistical Analysis

All values are expressed as mean ± SE. The statistical analysis was performed using GraphPad Prism™ (Version 4.00) software (GraphPad Software, Inc., San Diego, CA, USA). The one-way analysis of variance (one-way ANOVA), followed by Dunnett’s multiple comparison Post-Test, was used to analyze the difference between treatments. The concentration–response curves were constructed using the “Hill equation” according to previously published methods [26]. *p*-values < 0.05 and < 0.01 were considered as significant and very significant, respectively. 

## Figures and Tables

**Table 1:** IC50 values of the organic and hydroalcoholic extracts obtained from the June-harvested inflorescences of the different varieties of *Cannabis sativa* L. (*n* = 3) and the standard antioxidant agent Trolox in the antioxidant assays.

June-Harvesting *Cannabis sativa* L. Variety	Radical Scavenging Activity	
	DPPH	ABTS
	IC50 (CL) μg/mL	
Felina		
hydroalcoholic extract	183.8 (106.7–316.8) ^§§§,b^	80.56 (66.4–97.8) ^§§§,b^
organic extract	165.3 (134.7–202.9) °°°^,§§§,b,c^	18.6 (16.2–21.3) °°°^,§§§,b,c^
USO31		
hydroalcoholic extract	203.7 (182.2–227.7) ***	116.0 (98.1–137.1) *
organic extract	633.3 (525.0–764.0)	127.8 (96.5–169.3)
Ferimon		
hydroalcoholic extract	82.9 (69.3–99.0) ***^,§§§,a,b^	42.1 (20.8–85.6) ***^,§§§,a,b^
organic extract	237.90 (152.8–370.5) ^§§§,b^	76.8 (41.3–142.5) ^§§§,b^

* *p* < 0.05 and *** *p* < 0.001, significantly lower than the organic extract from the same cultivar in June (t-Student Test). °°° *p* < 0.001, significantly lower than the hydroalcoholic extract from the same cultivar in June (t-Student Test). ^§§§^ *p* < 0.001, significantly lower than the other cultivars in the same harvesting period (one-way ANOVA, followed by Bonferroni’s Multiple Comparison Post Test). ^a^ vs. Felina 32. ^b^ vs. USO 31. ^c^ vs. Ferimon.

**Table 2:** IC50 values of the organic and hydroalcoholic extracts obtained from the September-harvested inflorescences of the different varieties of *Cannabis sativa* L. (*n* = 3) and the standard antioxidant agent Trolox in the antioxidant assays.

September-Harvesting *Cannabis sativa* L. Variety	Radical Scavenging Activity	
	DPPH	ABTS
	IC50 (CL) μg/mL	
Felina		
hydroalcoholic extract	67.1 (48.5–92.7) ***^,§§§,b,c,d^	44.6 (37.1–53.6) ^§,b,d^
organic extract	143.5 (106.0–194.4) ^§§§,b,c,d^	11.9 (11.8–12.0) °°°^,§§§,b,c,d^
USO31		
hydroalcoholic extract	147.5 (110.4–197.0) ***^,§§§,d^	51.5 (42.8–62.0) **
organic extract	812.9 (732.9–901.7)	71.9 (64.1–80.6) ^§§§,c^
Ferimon		
hydroalcoholic extract	79.4 (73.5–85.8) ***^,§§§,b,d^	35.3 (20.4–61.2) ***^,§§§,a,b,d^
organic extract	704.7 (645.0–770.0) ^§§§,b^	123.5 (115.5–132.1)
Fedora		
hydroalcoholic extract	199.4 (149.8–265.3) ***	55.5 (50.5–60.9)
organic extract	427.0 (373.9–487.6) ^§§§,b,c^	27.5 (16.9–44.5) °°°^,§§§,b,c^

** *p* < 0.01 and *** *p* < 0.001, significantly lower than the organic extract from the same cultivar in September (t-Student Test). °°° *p* < 0.001, significantly lower than the hydroalcoholic extract from the same cultivar in September (t-Student Test). ^§^ *p* < 0.05 and ^§§§^ *p* < 0.001, significantly higher than the other cultivars in the same harvesting period (one-way ANOVA, followed by Bonferroni’s Multiple Comparison Post Test). ^a^ vs. Felina 32. ^b^ vs. USO 31. ^c^ vs. Ferimon. ^d^ vs. Fedora 17.

**Table 3:** Levels of the GSH and GSSG after treatment of the HepG2 cells with the positive control quercetin (50 µM), and the organic extracts of Felina 32, USO31 and Fedora from the September harvest and Ferimon from the June harvest (50 µg/mL). The GSH and GSSH were evaluated in cell lysates, calculated with respect to the corresponding calibration curves, and normalized with regard to cell viability. The data are mean ± SE from at least three independent experiments (*n* = 3).

Treatment	GSH	GSSG
	[µM]	
Control	69.6 ± 1.3	140.3 ± 1.3
Quercetin 50 µg/mL	236.3 ± 2.3 ***	158.9 ± 3.1 **
C. sativa Felina 32 50 µg/mL	197.0 ± 2.2 ***	164.4 ± 0.9 ***
C. sativa USO31 50 µg/mL	163.1 ± 1.3 ***	250.5 ± 2.9 ***
C. sativa Ferimon 50 µg/mL	79.3 ± 1.1 **	275.7 ± 2.3 ***
C. sativa Fedora 50 µg/mL	136.4 ± 1.7 ***	213.7 ± 2.2 ***

** *p* < 0.01 and *** *p* < 0.001 vs control (one-way ANOVA followed by Dunnett’s multiple comparison post test).

## Data Availability

Data are contained within the article.
